# Automated tracking of level of consciousness and delirium in critical illness using deep learning

**DOI:** 10.1038/s41746-019-0167-0

**Published:** 2019-09-09

**Authors:** Haoqi Sun, Eyal Kimchi, Oluwaseun Akeju, Sunil B. Nagaraj, Lauren M. McClain, David W. Zhou, Emily Boyle, Wei-Long Zheng, Wendong Ge, M. Brandon Westover

**Affiliations:** 1 Department of Neurology, Massachusetts General Hospital, Boston, MA USA; 2 Department of Anesthesia, Critical Care, and Pain Medicine, Massachusetts General Hospital, Boston, MA USA; 3 Department of Clinical Pharmacy and Pharmacology, University Medical Center Groningen, University of Groningen, Groningen, the Netherlands

**Keywords:** Diagnostic markers, Translational research, Disorders of consciousness, Diagnostic markers, Translational research

## Abstract

Over- and under-sedation are common in the ICU, and contribute to poor ICU outcomes including delirium. Behavioral assessments, such as Richmond Agitation-Sedation Scale (RASS) for monitoring levels of sedation and Confusion Assessment Method for the ICU (CAM-ICU) for detecting signs of delirium, are often used. As an alternative, brain monitoring with electroencephalography (EEG) has been proposed in the operating room, but is challenging to implement in ICU due to the differences between critical illness and elective surgery, as well as the duration of sedation. Here we present a deep learning model based on a combination of convolutional and recurrent neural networks that automatically tracks both the level of consciousness and delirium using frontal EEG signals in the ICU. For level of consciousness, the system achieves a median accuracy of 70% when allowing prediction to be within one RASS level difference across all patients, which is comparable or higher than the median technician–nurse agreement at 59%. For delirium, the system achieves an AUC of 0.80 with 69% sensitivity and 83% specificity at the optimal operating point. The results show it is feasible to continuously track level of consciousness and delirium in the ICU.

## Introduction

The “ICU triad” of pain, agitation, and delirium^1^ makes the intensive care unit (ICU) an intensely stressful experience for many critically ill patients. Sedatives and analgesics are widely used to minimize pain and agitation. Unfortunately, over- and under-sedation and analgesia are common, affecting about 70% of ICU patients.^2^ Over-sedation is associated with hypotension, prolonged ventilation, and ICU length of stay; under-sedation is likewise associated with pain, agitation, cardiac arrhythmias, immune dysfunction, and ventilator desynchrony. Both over- and under-sedation are associated with delirium, leading to poorer cognition^3^ and clinical outcomes.^4^

Many clinical assessment tools have been designed to monitor the level of consciousness in the ICU, including the Ramsay Scale,^5^ Sedation-Agitation Scale (SAS),^6^ and Richmond Agitation-Sedation Scale (RASS).^7^ Similarly, delirium is also assessed using behavioral scales such as the Confusion Assessment Method for the ICU (CAM-ICU)^8^ and Intensive Care Delirium Screening Checklist (ICDSC).^9^ The inter-rater agreement with these scales is relatively high.^7,8,10^ However, these assessments have inherent limitations: (1) they do not continuously track patient status; (2) they are not directly based on physiology; and (3) clinical assessments interrupt sleep. Some prior studies suggest that continuous tracking of the level of consciousness and delirium can contribute positively to ICU outcomes, and may improve ICU post-discharge outcomes and reduce costs.^11,12^

Various reviews^13–15^ have discussed the potential importance of continuous EEG (cEEG) monitoring in the ICU, such as recognizing non-convulsive status epilepticus, recognizing hypoactive delirium, and managing sedation levels. Although cEEG is increasingly implemented for monitoring patients after cardiac arrest and providing routine care in several hospitals, there are not enough trained clinical neurophysiologists available for reading EEG. Thus, tracking the level of consciousness and delirium in a continuous manner in the ICU has remained a challenge. Closely related to the topic, anesthesia depth monitors using EEG signals are used in the operation room to continuously track anesthesia depth, such as the Bispectral Index (BIS) (Aspect Medical Systems, Norwood, MA, USA) and Narcotrend Index (Monitor Technik, Bad Bramstedt, Germany). Their algorithms are mostly based on extracting various EEG spectral and entropy features and performing regression analysis.^16^ However, these monitors in the operation room are not optimized for the ICU, since they have been developed to monitor consciousness in relatively normal brains, while the brains in the ICU are very different.

Recent developments in deep learning have found promising applications in healthcare domains.^17^ Deep learning algorithms can learn task-relevant features from raw signals, reducing the need to handcraft features or biomarkers for a specific task. This ability is promising for EEG-based tracking of level of consciousness and delirium, where human experts may not be able to identify all features in EEG waveforms relevant to the brain states of interest.

Here we develop a deep learning model that automatically tracks both the level of consciousness and delirium. The input to the system is the preprocessed EEG waveform without extracting any features. We evaluate different aspects of its performance including tracking accuracy and delay. We interpret the model by showing the important regions of the EEG signal that lead to the final prediction. The results provide evidence for the feasibility of continuously tracking level of consciousness and delirium in the ICU.

## Results

### Tracking level of consciousness

**Fig. 1:**
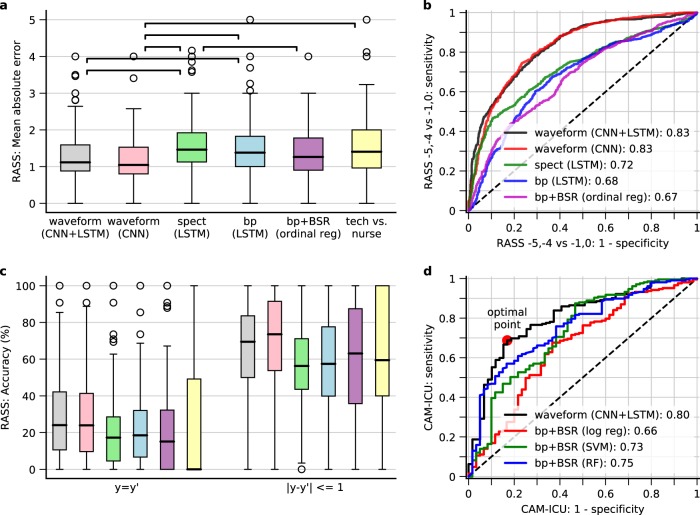
Tracking performance for level of consciousness and delirium. **a** Boxplot of the mean absolute error (MAE) between true and predicted RASS levels per patient in the pooled testing sets from all tenfolds. The upper, middle line, and bottom of the boxes are the 75% (Q3), 50% (median), 25% (Q1) percentiles, respectively. The whiskers extend to the smallest and largest value which is within [Q1–1.5 × IQR, Q3 + 1.5 × IQR]; circles are the outliers outside this range. The horizontal bars with stars indicate pairs with significantly different medians (*p* < 0.05). spect spectrogram, bp band power, BSR burst suppression ratio, tech research technician. **b** Receiver operator curves (ROC) when only focusing on RASS −5, −4 vs. −1, 0. The *x*-axis is the false-positive rate. The *y*-axis is the true positive rate. **c** Accuracy when allowing 0 (left) and 1 (right) level difference between true and predicted RASS. **d** ROC for detecting delirium. The markers indicate the optimal operating point according to different criteria as shown in Table 1

As shown in Fig. 1a, CNN + LSTM achieved similar MAE compared to using CNN (*p* = 1.0), and outperformed spectrogram or band power (*p* < 0.05), suggesting that CNN learned a better set of features compared to spectral domain only. The MAE was comparative to technician-nurse agreement. In Fig. 1b we compared the AUCs for RASS assessments only at −5, −4 vs. −1, 0. CNN + LSTM achieved AUC 0.83 (95% CI 0.81–0.85). CNN + LSTM had significantly better AUC than the non-deep learning method (bp + BSR: using band power and burst suppression ratio (BSR) as the features and ordinal regression as the model). As shown in Fig. 1c, the accuracy was 24% for the CNN + LSTM model which was higher than technician–nurse agreement (*p* < 0.05); when allowing for one level of difference, the accuracy was 70% which was comparative to technician–nurse agreement. Overall, CNN + LSTM and CNN only achieved the best performance. LSTM mainly learned to smooth without reducing performance. The distributions of the individual performance metrics for CNN + LSTM are shown in Supplementary Fig. 4.

### Tracking delirium

In Fig. 1d we show the receiver operator curve (ROC) between true CAM-ICU and the predicted probability of having positive CAM-ICU. It achieved an AUC of 0.80 (95% CI 0.73–0.86). In Table 1 we show the sensitivity, specificity, and thresholds at various operating points. The optimal operating point^18^ is associated with sensitivity of 0.69 and specificity of 0.83 at a threshold of 0.59.

**Table 1:** Sensitivity, specificity, and threshold at different operating points for tracking delirium

Condition	Sensitivity	Specificity	Threshold
Sensitivity at predefined values	0.6	0.86	0.69
	0.7	0.81	0.58
	0.8	0.64	0.37
	0.9	0.37	0.09
Specificity at predefined value	0.86	0.6	0.21
	0.77	0.7	0.41
	0.70	0.8	0.58
	0.55	0.9	0.78
Optimal point	0.69	0.83	0.59

A probability calibration curve for delirium detection is shown in Supplementary Fig. 5, including the curve before and after re-calibration. The calibration error (mean absolute error to the diagonal line) after re-calibration is 0.040 (95% CI 0.032–0.094).

### Tracking delay for level of consciousness

**Fig. 2:**
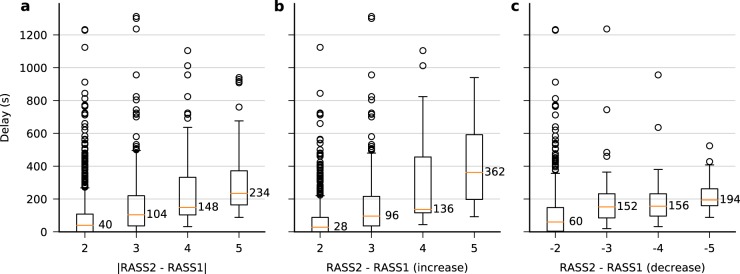
Tracking delay for level of consciousness. **a** Boxplot of the delays in seconds from the transition point to the point where the prediction matches the new RASS level to within one level difference. It includes both transitions to higher and lower RASS levels. **b** Transitions to higher RASS levels. **c** Transitions to lower RASS levels. In all panels, the upper, middle line, and bottom of the boxes are the 75% (Q3), 50% (median), and 25% (Q1) percentiles respectively. The whiskers extend to the smallest and largest value which is within [Q1–1.5 × IQR, Q3 + 1.5 × IQR]; circles are the outliers outside this range

In Fig. 2 we show boxplots for several different cases. The delay was longer for larger increases or decreases in the level of consciousness, since it required longer time for the “*z*-score” (the output from the final ordinal regression layer before applying the thresholds to convert into discrete RASS levels, see Methods) to climb up or drop down for a larger distance.

### Example of continuous tracking

**Fig. 3:**
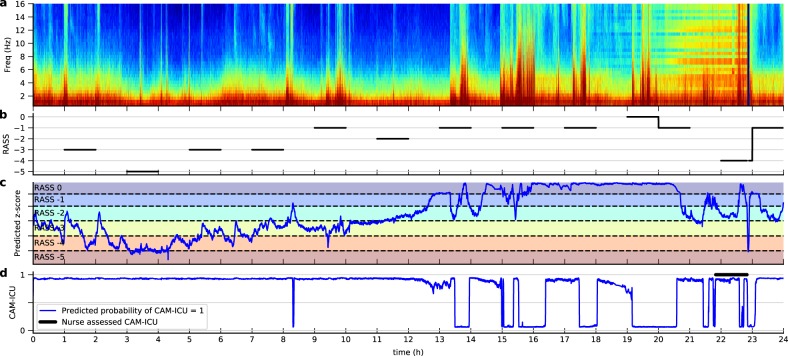
Example of the model output on 24 h of continuous EEG. **a** EEG spectrogram. **b** RASS levels assessed by ICU staff. The black line indicates the period 30 min before and after each assessment. **c** Predicted *z*-score of the level of consciousness (blue line). Dashed lines are the thresholds learned by the model to partition the *z*-score into discrete RASS levels. The *z*-score beyond the top or bottom dashed line is log-transformed to suppress large values for visual purpose. **d** CAM-ICU score assessed by ICU nurse staff (black line) and the predicted probability of CAM-ICU = 1 (blue line)

The model was trained only on EEG data 1 h around each assessment to maintain proximity to RASS or CAM-ICU scores. Here we illustrate the application of the model on longer, continuous EEG signals, as shown in Fig. 3. For level of consciousness, we show the predicted *z*-score, which is a continuous value but can be discretized into RASS levels. The predicted *z*-score matched well with the periods around RASS assessments (solid lines in panel b). Panel d shows the continuously tracked probability of delirium.

### Model interpretation

**Fig. 4:**
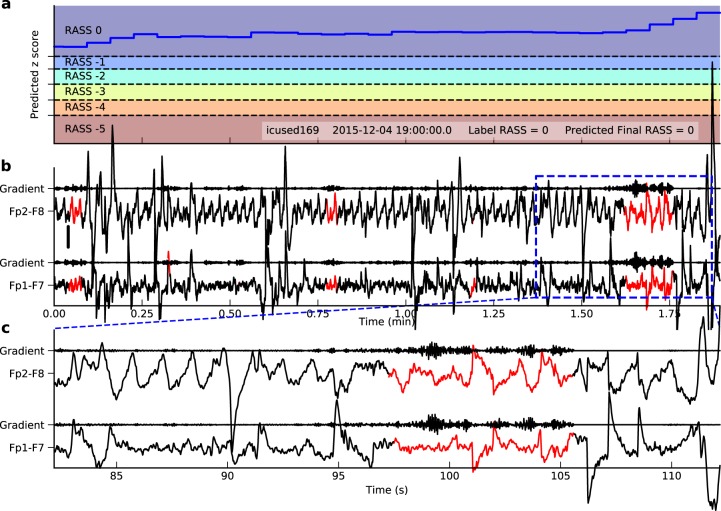
Interpretation of EEG patterns learned by the model for RASS 0 based on gradient information. **a** Trace of the predicted *z*-score and the thresholds to discretize it to RASS levels. Here the predicted RASS is always 0. **b** The corresponding signal and the gradient of the final *z*-score with respect to the signal. The red parts have gradient larger than the 95% percentile of the gradient in the segment. Time is in minutes. The scale is shown on the left middle side representing 50 µV. **c** A zoomed view of the blue dashed rectangle. Here time is in seconds. The EEG segment in red shows lateral eye movements, suggesting the patient is awake and looking around

**Fig. 5:**
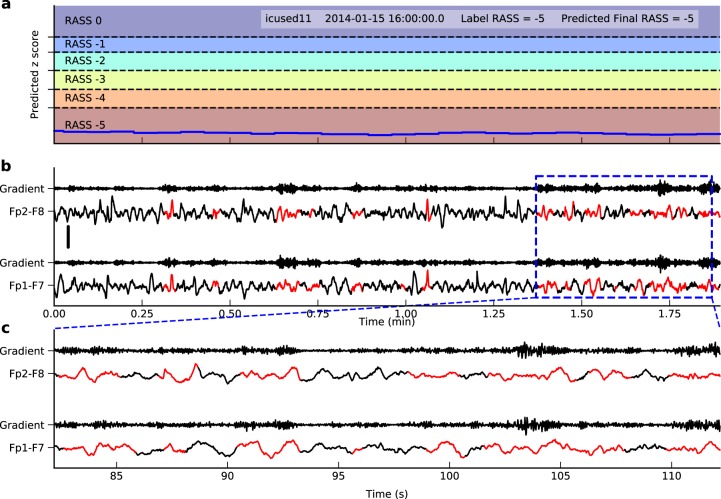
Interpretation of EEG patterns learned by the model for RASS −5 based on gradient information. The panels are similar to those in Fig. 4. The EEG shows low amplitude slow waves, characteristic of coma. The scale is shown on the left middle side of panel **b** representing 50 µV

To interpret what EEG patterns this model has learned, we computed the gradient of the *z*-score at the end of a period with respect to the input EEG signal. The signal parts with larger gradient had higher impact on the final *z*-score, and were thus more important to the model predictions. Figure 4 shows a 1.9-min signal with both true and predicted RASS at 0, i.e. awake and calm state. The important parts (red) showed blinking artifacts, a sign of wakefulness. In Fig. 5 we demonstrate another example with both true and predicted RASS at −5, i.e. coma. Here the important parts (red) showed slow waves and low amplitude, which are characteristic of depressed levels of consciousness (e.g. sleep, encephalopathy, and coma).

## Discussion

We have demonstrated a system that can automatically track both the level of consciousness (LOC) and delirium in the ICU using frontal EEG signals and deep learning. The results show the feasibility of providing continuous measures of level of consciousness and delirium for ICU patients. Unlike current behavioral assessments, our model is based on physiological signals, being more direct than behavior. The values also have natural interpretations: for level of consciousness, the discretized levels map onto RASS scores; for delirium, the predicted value between 0 and 1 is the probability of being delirious. This system has the potential to improve the management of both sedation and delirium in ICU.

Multiple studies have studied the classification of level of consciousness in both operative room and ICU patients. Engemann et al.^19^ used the extra-trees algorithm to classify 327 patients with unresponsive/minimally conscious state vs. 66 healthy controls. They applied the classifier across different cohorts, EEG protocols and centers, and obtained AUC ranging from 0.73 to 0.78. The modest performance confirms the heterogeneity between cohorts, protocols and centers. Nagaraj et al.^20^ used atomic decomposition and a support vector machine classifier to classify RASS −5, −4 vs. −1, 0 based on 44 patients—a subset of our dataset. They achieved AUC at 0.91. The results are comparable to the case of training specifically a binary classification achieving AUC 0.89 (95% CI 0.88–0.91) instead of ordinal regression (Supplementary Fig. 6). As discussed in their Dataset Section, they excluded difficult cases and used two RASS assessments from each patient. Our results are more generalizable by including more patients and achieving similar performance.

Multiple studies have compared simple features of EEG signals in delirium vs. non-delirium patients, such as spectral power^21^ and functional connectivity,^22^ anticipating the possibility of continuously tracking delirium from EEG in ICU.^23^ Our model provides the ability to track delirium in continuously with similar AUC as clinical risk prediction models.^24,25^ van der Kooi et al.^26^ describe delirium detection based on EEG collected from 28 delirious and age-gender matched 28 non-delirious post-cardiothoracic surgery patients. They found that the relative delta power at F8-Pz channel during the eye closed condition is increased in delirium patients, and achieves the best discrimination with AUC 0.99 (95% 0.97–1.00). Of note, our study is carried out in mechanically ventilated and sedated patients in the ICU, which represents a more severely ill population than post-operation patients.

Our model exhibits delay in response to the change in level of consciousness. The change in the predicted value lagged behind the change in EEG by 0.5–6 min. Although not directly comparable, Zanner et al.^27^ measured the time delay in BIS, Narcotrend Index, and cerebral state index when measuring anesthesia depth in the operating room. They found that 24 to 122 s were needed before the new state was identified. Similarly, their time delays were not constant and varied depending on the starting and ending anesthesia depth. For tracking level of consciousness and delirium in ICU patients, these delays are acceptable in practice.

The tracking performance for level of consciousness is not perfect. As seen in Fig. 1c, the exact agreement between the true and predicted RASS is low at 24% for using CNN + LSTM. When allowing one RASS level difference, the agreement increases up to 70%. The discrepancy probably comes from multiple sources. (1) The behavioral difference between consecutive RASS levels in terms of EEG is small. In fact, RASS −1 is defined as eye contact more than 10 s, while RASS −2 is defined as less than 10 s. This distinction may not be possible to accurately sort out in terms of brain activity. (2) Heterogeneity exists between patients, such as different ICU admission diagnoses and different sedatives and analgesics,^29^ each of which may have a different effect on EEG. Multiple different EEG patterns may therefore correspond to the same behavioral state and thus to the same RASS level. For example, non-convulsive seizures and burst suppression can both present clinically with coma, but have very different EEGs. The results highlight the difficulty of inferring a behavioral state (RASS) from the EEG—more difficult than in the operating room (e.g. BIS). A future direction is to calibrate the prediction algorithm based on the existing RASS + EEG observations for a given patient. (3) We have not considered the differences between effects of various sedative and analgesic drugs in the ICU. (4) Human error and variability are inherent in each clinical assessment. We have measured the technician-nurse agreement in our dataset (Fig. 1, Supplementary Fig. 8). The median of the mean absolute difference between technician and nurse assessments across all patients is between 1.00 and 1.97 RASS levels. Even though formal studies show good between-rater agreement,^7^ our data show that in practice the agreement is not as high.

There are some limitations in our approach. (1) Our sample size for RASS measurement was 174 patients, which may not be large enough to capture the full range of variation in EEG patterns and corresponding behavioral states that occur in the ICU. (2) Due to heterogeneity between patients, the variance of tracking performance among patients is large. More detailed stratification of patients into different phenotypes and training different models for each phenotype is a possible future approach. (3) The current model does not consider the different effects of different sedatives on the EEG. Our model likely mainly reflects EEG patterns under propofol, given that patients in our cohort were mainly sedated using propofol. (4) Our data include no positive RASS scores during times when both CAM-ICU and EEG are available, as shown in Supplementary Fig. 1b. We speculate that there are two likely reasons for this. First, hypoactive delirium is more common than hyperactive delirium, particularly in ICU patients. Second, nurses actively adjust sedation levels to prevent patients becoming agitated or combative while patients remain on mechanical ventilation, and in our study the EEG leads were removed when the patient was weaned from mechanical ventilation. Thus the potential utility of EEG for hyperactive delirium has to be studied in other cohorts. (5) CAM-ICU assessments (where incidence is high) were performed only done once per day. More frequent assessment of delirium status would better reflect the dynamic course of delirium and provide more training data.

## Methods

### Dataset

The study was a single-center, prospective observational study approved by the Partners Institutional Review Board (IRB). The IRB waived the requirement for written signed consent in this study. The EEG signals were collected from 195 distinct ICU patients. The inclusion criteria were: (1) age ≥18 years; (2) on mechanical ventilation; and (3) have at least one RASS or CAM-ICU assessment during EEG recording. The exclusion criteria were: (1) any known focal neurologic deficits or dementia; and (2) poor EEG signal quality by visual inspection (ten patients excluded). The final dataset contains 174 patients. The average ICU stay was 12–13 days. The most commonly used sedative was propofol. Patient characteristics are summarized in Table 2.

**Table 2:** Patient characteristics

Characteristics	For RASS	For CAM-ICU
Number of patients	174	121
Number of assessments	3366	258
RASS: *N* (% among all assessments), only included non-positive RASS assessments		
0	355 (10.5%)	33 (12.8%)^a^
−1	696 (20.7%)	34 (13.2%)
−2	660 (19.6%)	44 (17.1%)
−3	848 (25.2%)	67 (26.0%)
−4	621 (18.4%)	27 (10.5%)^b^
−5	186 (5.5%)	53 (20.5%)^b^
CAM-ICU: *N* (% among all assessments)		
0	–	60 (23.2%)
1	–	199 (76.8%)
Age: year, median (IQR)	61 (51, 70)	61 (51, 70)
Male: *N* (%)	117 (67%)	84 (69%)
Race: *N* (%)		
White	154 (89%)	105 (86%)
Black or African	9 (5.2%)	7 (5.7%)
Asian	2 (1.1%)	2 (1.6%)
More than one race	1 (0.6%)	1 (0.8%)
Unknown	8 (4.6%)	7 (5.7%)
BMI: kg/m^2^, median (IQR)	29 (24, 35)	29 (23, 35)
Days in ICU: day, median (IQR)	12 (7, 20)	13 (8, 20)
APACHE II at ICU admission: median (IQR)	23 (15, 28)	23 (15, 28)
CCI at ICU admission: median (IQR)	3 (2, 5)	3 (2, 5)
ICU admission diagnosis: *N* (%), showing more than ten patients for either RASS or CAM-ICU		
Acute respiratory failure	111 (64%)	77 (63%)
Kidney failure, or chronic kidney disease	51 (29%)	34 (28%)
Surgery (including gastrointestinal)	43 (25%)	30 (25%)
Diabetes mellitus	39 (22%)	30 (25%)
Chronic obstructive pulmonary disease	31 (18%)	21 (17%)
Arrhythmia, congestive heart failure, myocardial ischemia	27 (16%)	19 (16%)
Sepsis	26 (15%)	19 (16%)
Liver disease or failure	25 (14%)	17 (14%)
Peripheral vascular disease	14 (8%)	6 (5%)
Solid tumor	14 (8%)	11 (9%)
Average infusion rate: mg/h/kg (number of patients receiving each drug, percentage)		
Propofol	2.03 (132, 76%)	1.76 (73, 60%)
Hydromorphone	0.04 (63, 36%)	0.04 (29, 24%)
Dexmedetomidine	0.001 (34, 20%)	0.001 (18, 15%)
Fentanyl	0.0023 (21, 12%)	0.001 (10, 8%)
Ketamine	1.00 (9, 5%)	1.17 (5, 4%)
Midazolam	0.13 (9, 5%)	0.17 (4, 3%)

^a^The last column shows the RASS assessed during CAM-ICU

^b^CAM-ICU is unavailable for RASS −5 and −4. We treat them as CAM-ICU = 1

### RASS and CAM-ICU

To measure the level of consciousness, we used the Richmond agitation-sedation scale (RASS)^7^ as the target to train the model. RASS was assessed by ICU nurses and clinical research technicians approximately every 2 h. RASS has ten levels from −5 to +4 as shown in Supplementary Table 1. The range from −5 to 0 (inclusive) describes different levels of sedation, where −5 and −4 indicate coma (unarousable, no response to verbal or noxious stimulation) and 0 indicates an alert and calm state. The range from +1 to +4 (inclusive) describes different levels of agitation which are associated with hyperactive delirium. In this study we limited RASS assessments to those of normal or decreased levels of arousal only, i.e. −5 to 0, since (1) there was no positive RASS during CAM-ICU assessments with EEG signal available in the dataset (Supplementary Fig. 1) and (2) being combative and agitated can be reliably detected by ICU staff.

To measure delirium, we used the CAM-ICU as the target to train the model. The CAM-ICU is a screening protocol that is performed about every 24 h^8^ (Supplementary Table 2). While unresponsive patients (RASS = −4 or −5) are typically not further assessed in formal use of the CAM-ICU, we treated these patients as CAM-ICU positive for model training purposes, given the clearly abnormal mental status.

### EEG preprocessing

The EEG signals were recorded using Sedline brain function monitors (Masimo Corporation, Irvine, CA, USA), with 250 Hz sampling rate and 4 frontal electrodes. We re-referenced the signals to 2 bipolar channels: Fp1-F7 and Fp2-F8. The signals were first notch filtered at 60 Hz, then bandpass filtered between 0.5 Hz to 20 Hz, and finally downsampled to 62.5 Hz.

We took 1 h EEG segments 30 min before and 30 min after each RASS or CAM-ICU assessment. This is because the assessment times recorded by the ICU nurses may be imprecise, since they are recorded after performing assessments. We therefore included the longer EEG segment to ensure it included the actual assessment time.

EEG artifact were defined based on the presence of any of the following in any EEG channel: (1) maximum amplitude higher than 1000 µV; (2) standard deviation less than 0.2 µV; (3) overly fast changes of more than 900µV within 0.1 s; or (4) spuriously staircase-like spectrum, when the maximum value obtained by convolution with a predefined staircase-like kernel exceeds an empirical threshold of 10, indicating the presence of nonphysiologic single-frequency artifacts from ICU machines (e.g. cooling blankets or pumps).

### Deep learning model

The overall deep learning model consisted of convolutional neural network (CNN) followed by long-short term memory (LSTM), as shown in Supplementary Fig. 9. CNN extracts useful information from each 4 s in the EEG waveform and LSTM provides the temporal context. The CNN followed the architecture in Hannun et al.^28^ It contains 8 blocks mainly consisting of two convolutional layers (conv) and a skip layer maxpooling connection. The output from CNN is then fed to a two-layer LSM, followed by an output layer, which is ordinal regression for RASS and binary classification for CAM-ICU. The ordinal regression learns a continuous “*z*-score” and the thresholds. If needed, we can apply the learned thresholds to discretize *z*-score into RASS levels. The binary classification outputs the probability of being CAM-ICU positive (delirium). The detailed description of the model architecture and coding details can be found in Supplementary Methods.

### Model training

To avoid the model being overfit to the dataset, we randomly split patients into ten groups (folds). We took each fold as a testing set, and the other ninefolds as the training set. For the training set we further randomly selected 10% of assessments as the validation set, and the remaining 90% of assessments as the training set. The model with the minimum loss on the validation set was used, and then results were calculated for the held-out testing set. The above procedure was repeated for each fold.

To prepare data for CNN, the 1 h EEG signal around each assessment was segmented into 4 s windows with 2 s overlap (Supplementary Fig. 2a). We removed 4s-segments identified as artifact. 10% of segments were removed due to artifacts. The input to the CNN has size N x 2 × 250, where N is the number of 4s-segments, 2 is the number of channels and 250 is the number of time points in 4 s (62.5 Hz). The choice of 4 s window is inspired by domain knowledge – in clinical neurology practice, windows of 10 s are used, but 4 s is enough to discern features usually used to describe the EEG, e.g. the presence of delta or theta slowing, epileptiform abnormalities, and EEG suppression.

Data preparation for LSTM is different. There are 900 4s-segments in each 1 h EEG signal. Training an LSTM model on such a long sequence is difficult. Therefore we trained the two layers of LSTM separately while fixing the parameters in the already trained CNN. The first LSTM layer was trained using 9.5 min sequences with step size 1 min (Supplementary Fig. 2b). The input had size N x 142 × 2 × 250, where 142 is the number of 4s-segments in a 9.5 min sequence. To train the second LSTM layer, we fixed the first LSTM layer. 1 h sequences were used with size N x 900 × 2 × 250, where 900 is the number of 4s-segments in a 1 h sequence (Supplementary Fig. 2c). Sequences with more than 50% of 4s-segments being artifact were removed, otherwise the artifacts in 4s-segments were kept to ensure continuity of the sequence. 9% of the sequences were removed.

For the CAM-ICU, since the number of samples was less than that of RASS, we copied the first M layers of the RASS CNN model to the CAM-ICU CNN model and fixed them to avoid overfitting; only the layers after the first M layers were trained. The performance of different M’s is shown in Supplementary Fig. 3. Here we took M = 5 since it achieved the best validation performance.

In both tasks, to address the imbalance of RASS levels or CAM-ICU scores in the dataset, we computed sample weights for each level inversely proportional to the number of examples in this level from the training set. The models were trained with a minibatch size of 32 and the RMSprop optimizer with learning rate 0.001.

### Model evaluation

The final performance was reported using the testing patients pooled from all folds. For tracking RASS, the predicted *z*-score was averaged across all 4s-segments in each 1 h sequence, and then the thresholds learned by the ordinal regression layer were used to discretize the averaged *z*-score to produce the predicted RASS level. We evaluated the RASS tracking performance using three metrics: (1) balanced mean absolute error (MAE), i.e. the average absolute difference between true and predicted RASS levels, weighted by class weights inverse proportional to number of samples in that class; (2) balanced accuracy when allowing up to one level difference, weighted by class weights; and (3) binary classification performance, measured by area under the receiver operator curve (AUC), for discriminating RASS levels −5 or −4 (“coma”) from −1 or 0 (“awake”), while discarding other levels. For tracking CAM-ICU, the predicted probability was averaged across all 4s-segments in each 1 h sequence to get the probability of being delirious.

The accuracy per 4 s without averaging (CNN only) is shown in Supplementary Fig. 7. These accuracies are worse than the averaged versions. The 4 s window is best thought as a step for local evaluation of the signal, and these local evaluations are aggregated to compute the probability of RASS/delirium at the present time, based on the prior EEG. Our model still reports an updated prediction every 4 s (this is the step size), although the prediction for the present time is based on the past 1 h. By contrast, in the ICUs in our institution, RASS is manually assessed every 2 h, and delirium is formally assessed only one time per day, thus the proposed method is an improvement.

### Technician–nurse agreement

Since RASS assessments were available from both ICU nurses and clinical research technicians, we were able to measure the technician–nurse agreement, as follows. For each assessment done by each research staff member, we found the closest nurse assessment for the same patient. We excluded assessment pairs more than 4 h apart.

### Baseline methods to be compared

To compare with other deep learning candidates, we built three other models (1) using EEG waveforms as input and CNN only; (2) using EEG spectrograms as input and LSTM only; and (3) using EEG band powers as input and LSTM only. The CNN and LSTM had the same structure as in Supplementary Fig. 9. The EEG band powers included delta (0–4 Hz), theta (4–8 Hz), and alpha (8–12 Hz), as well as the relative band power normalized by total power (0–12 Hz).

To compare with non-deep learning methods, we extracted the above band power from each 4s-segment, which were then averaged across 1 h time. We also extracted the BSR, i.e., the proportion of time within 1 h having signal envelope less than 5 µV. After generating these features, we trained ordinal regression for RASS; and logistic regression, support vector machine, and random forest for CAM-ICU.

### Statistical tests

To compare the performance among multiple algorithms, we used Kruskal–Wallis one-way analysis of variance (KW-ANOVA), which is a nonparametric version of ANOVA. The null hypothesis is that the medians of all groups are equal. We used Dunn’s test (two-sided) as the post hoc test together with Bonferroni multiple comparisons correction to decide which pairs had significantly different medians. The confidence intervals mentioned below are all 95% confidence interval obtained by bootstrapping 1000 times.

### Delays in tracking level of consciousness

For each patient we artificially concatenated two segments of 9.5 min EEG signals with different RASS levels, denoted as RASS1 and RASS2, where the absolute difference between RASS1 and RASS2 was more than one level. The delay is defined as the time from concatenation point to the first time the prediction reaches RASS2 ± 1.

### Reporting Summary

Further information on research design is available in the Nature Research Reporting Summary linked to this article.

## Supplementary information

Supplementary Material

reporting summary

## Data Availability

The data that support the findings of this study are available from the corresponding author upon reasonable request.

## Supplementary information

**Supplementary information** accompanies the paper on the *npj Digital Medicine* website (10.1038/s41746-019-0167-0).

## Code Availability

Code for the algorithm development, evaluation, and statistical analysis is open source with no restrictions and is available from https://github.com/mghcdac/rass_delirium_eeg_prediction.
