# Supplementary material for: Automated tracking of level of consciousness and delirium in critical illness using deep learning
Source: NPJ Digit Med. 2019 Sep 9;2:89. doi: 10.1038/s41746-019-0167-0 (PMC6733797; doi:10.1038/s41746-019-0167-0)
Supplement: Supplementary file 1 — Supplementary Material [file 41746_2019_167_MOESM1_ESM.pdf]

Supplementary Materials for
"Automated Tracking of Level of Consciousness and Delirium in Critical Illness using Deep Learning"

Haoqi Sun, PhD[1], Eyal Kimchi, MD, PhD[1], Oluwaseun Akeju, MD[2], Sunil B. Nagaraj, PhD[3], Lauren M. McClain, BA[1], David W. Zhou, MSc[2], Emily Boyle, BSc[1], Wei-Long Zheng, PhD[1], Wendong Ge, PhD[1], M. Brandon Westover, MD, PhD[1]

[1] Department of Neurology, Massachusetts General Hospital, Boston, MA, USA

[2] Department of Anesthesia, Critical Care, and Pain Medicine, Massachusetts General Hospital, Boston, MA, USA

[3] Department of Clinical Pharmacy and Pharmacology, University Medical Center Groningen, University of Groningen, The Netherlands

Supplementary Methods

The overall deep learning model consisted of a feed-forward network implemented using convolution neural network (CNN) for extracting features from the EEG waveform, followed by a recurrent neural network (RNN) implemented using long-short term memory (LSTM) for providing the temporal context from subsequent segments. The convolution layers use filters to "scan" the EEG waveform, if the EEG looks like a filter, the convolved value is large. There are multiple filters per convolution layer, each "scanning" for a different pattern. Max-pooling is a process of down-sampling the output of the convolution layer by extracting the maximum convolved value in a given segment of data.

The CNN contains an initial convolution layer and 8 resblocks. Each resblock consists of two batch normalization layers, two leaky rectified linear units (leaky ReLU), two dropout layers, two convolution layers, and a max-pooling layer if required to subsample the input. The max-pooling layer is on the residual connection (skip layer connection). The network increases its number of filters by 32 for every 4 resblocks, and subsamples the signal 4 times for every 2 resblosks. Therefore, the sequence of output size of each hidden layer is: (2 x 250) (this is the input EEG size with 2 channels and 4 seconds, 62.5Hz) → (32 x 250) (after the initial convolution layer) → (32 x 63) → (32 x 63) → (32 x 16) → (64 x 16) → (64 x 4) → (64 x 4) → (64 x 2) → (96 x 2). Finally it is flattened to be (192). If CNN is used as a feature extractor for LSTM, these 192 features are fed to the LSTM; otherwise the features are fed to an output layer. The LSTM has 2 layers with 16 and 4 hidden nodes in each layer for RASS; and 8 and 4 hidden nodes for CAM-ICU.

The output layer for RASS is an ordinal regression layer, implemented using ordistic regression (ordinal generalization of logistic regression)[1] to learn both the weights and thresholds. The output of the ordinal regression was a continuous "z-score", and if needed, we applied the learned thresholds to discretize it into RASS levels. The output layer for the CAM-ICU was logistic regression since it is a binary

classification problem. The LSTM outputs a number for each 4s window. We take the average of these numbers across the 1h as the final output.

We used PyTorch (https://pytorch.org/), the community-based open-source deep learning platform, written in Python (https://www.python.org/), as our coding tool. The results are generated using a desktop computer with 64GB memory, 24 CPUs and 4 Nvidia GTX 1080 Ti GPUs with 11GB memory each. The code that support the findings of this study are available from the corresponding author upon reasonable request.

Supplementary Figures

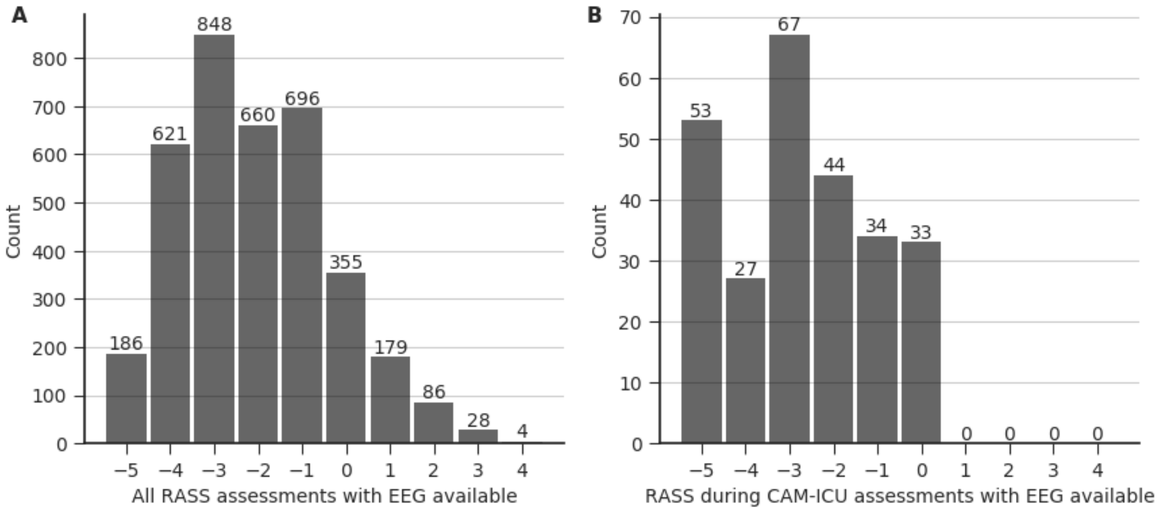

Supplementary Figure 1. The distribution of RASS scores in (A) All assessment with EEG available. (B) CAM-ICU assessments with EEG available.

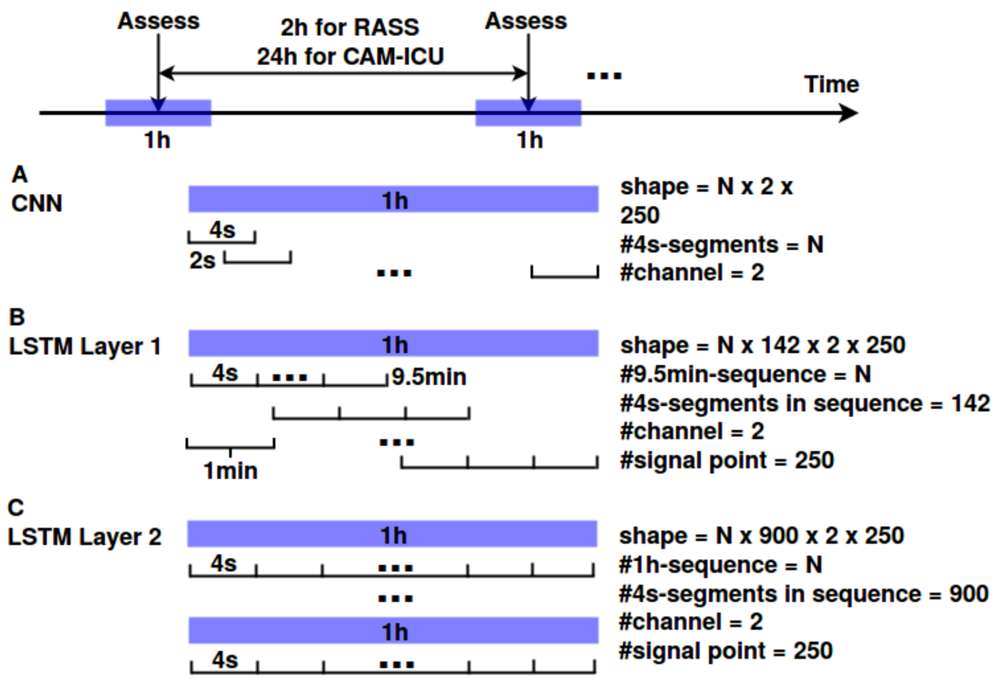

Supplementary Figure 2. The illustration of data preparation for training the model. (A) for training the CNN network; (B) for training the first layer of LSTM; and (C) for training the second layer of LSTM.

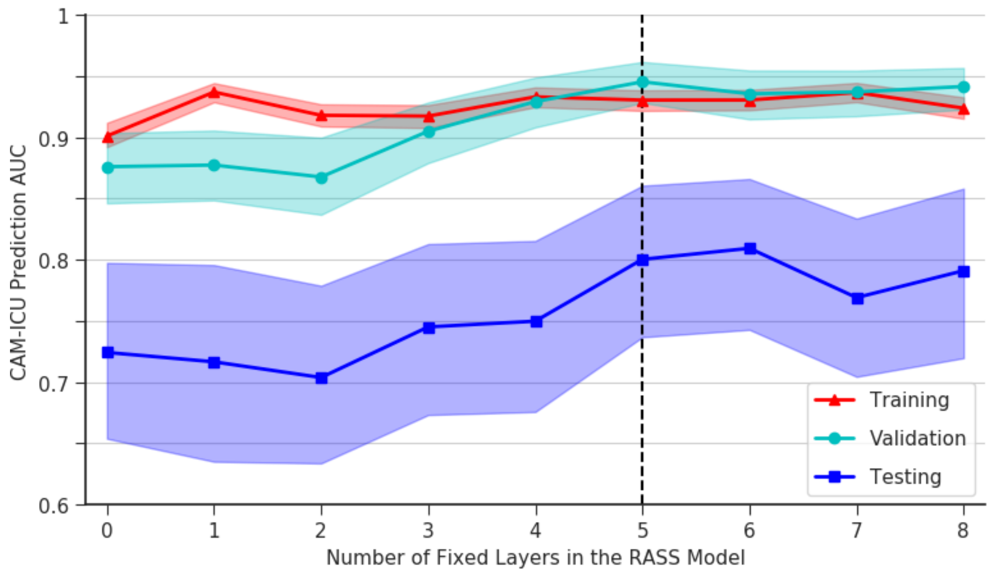

Supplementary Figure 3. The training, validation and testing AUC for CAM-ICU prediction when fixing different number of layers in the CNN model from RASS prediction. The dashed vertical line shows that when fixing the first 5 layers, the model has the maximum validation AUC, therefore we should use this setting.

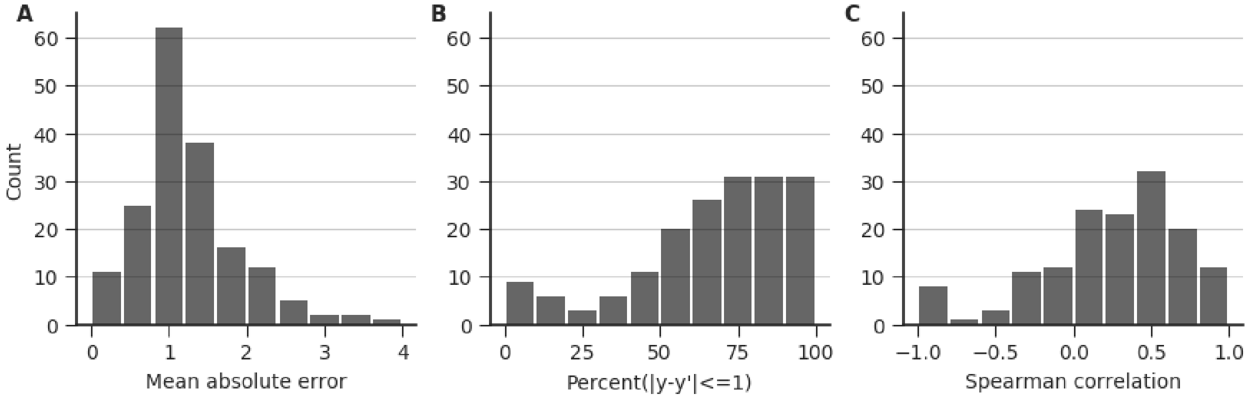

Supplementary Figure 4. **(A)** Histogram of the mean absolute error across all patients from the testing sets of all 10 folds. **(B)** Histogram of the percentage of agreement when allowing 1 level error in the prediction. **(C)** Histogram of the Spearman correlation between the true RASS and the predicted z-score.

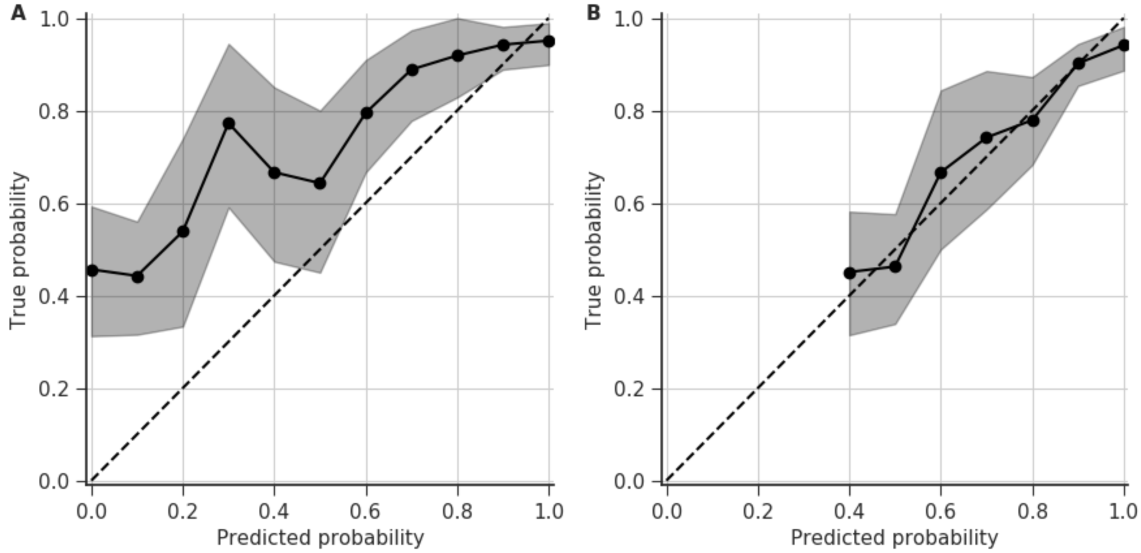

Supplementary Figure 5. Calibration curve for delirium. (A) The x-axis is the predicted probability. The y-axis is the probability of true CAM-ICU=1 when their predicted probability is within the range of predicted probability ±0.1. The calibration error is defined as the mean absolute difference compared to the diagonal perfect line. The 95% confidence band is obtained by bootstrapping 1000 times. The calibration error is 0.24 (95% CI 0.18 – 0.30). The calibration can be improved as in (B) by doing re-calibration, which learns an optimal transformation so that the transformed predicted probability achieves minimum calibration error. The calibration error after re-calibration is 0.040 (95% CI 0.032 – 0.094).

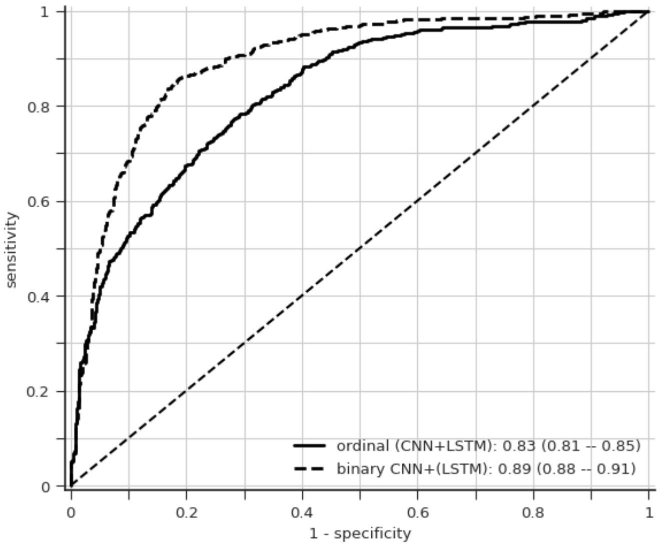

Supplementary Figure 6. (Solid line) The AUC between true RASS (binary, -5, -4 vs. -1, 0) and the predicted probability of being RASS -1 or 0 obtained by ordinal regression, using only assessments with RASS -5, -4, -1, 0. (Dashed line) Similar, but the predicted probability is obtained by training a binary classifier. As expected, binary classification achieves higher AUC than ordinal regression (p<0.05 by bootstrapping 1000 times), since binary classification focuses on the separation between RASS -5, -4 vs. -1, 0; while ordinal regression focuses on the relative ordering of all RASS levels.

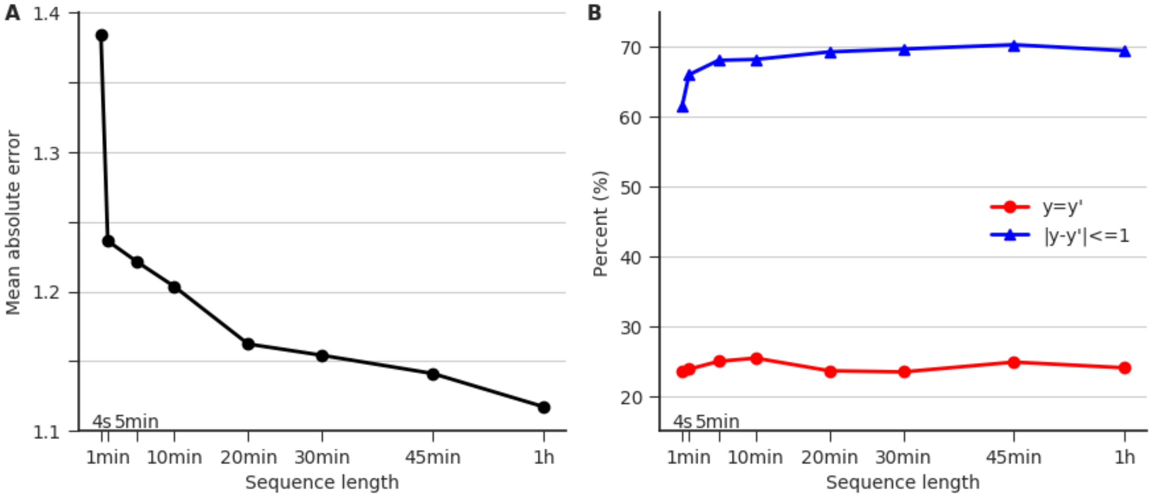

Supplementary Figure 7. The testing performance for different sequence lengths. The y-axis shows the median across all patients. The leftmost point at 4s is the performance when 4s-segments are fed to CNN without LSTM without any averaging of the final result.

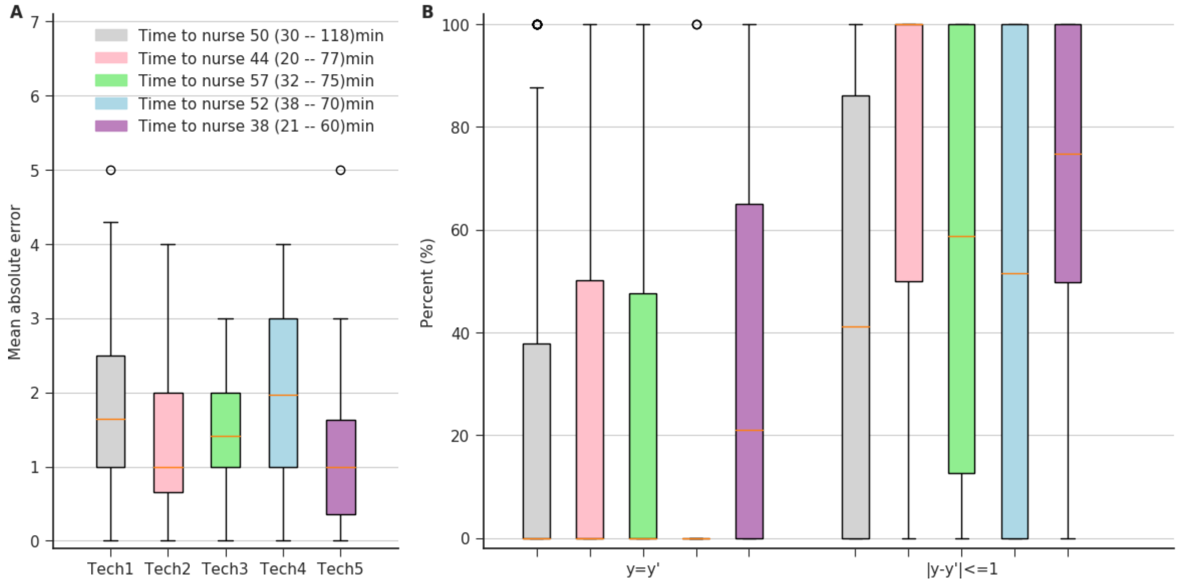

Supplementary Figure 8. (A) The boxplot of the mean absolute error for each clinical research technician vs. nurse per patient. (B) The boxplot of the accuracy when allowing up to 1 RASS level difference.

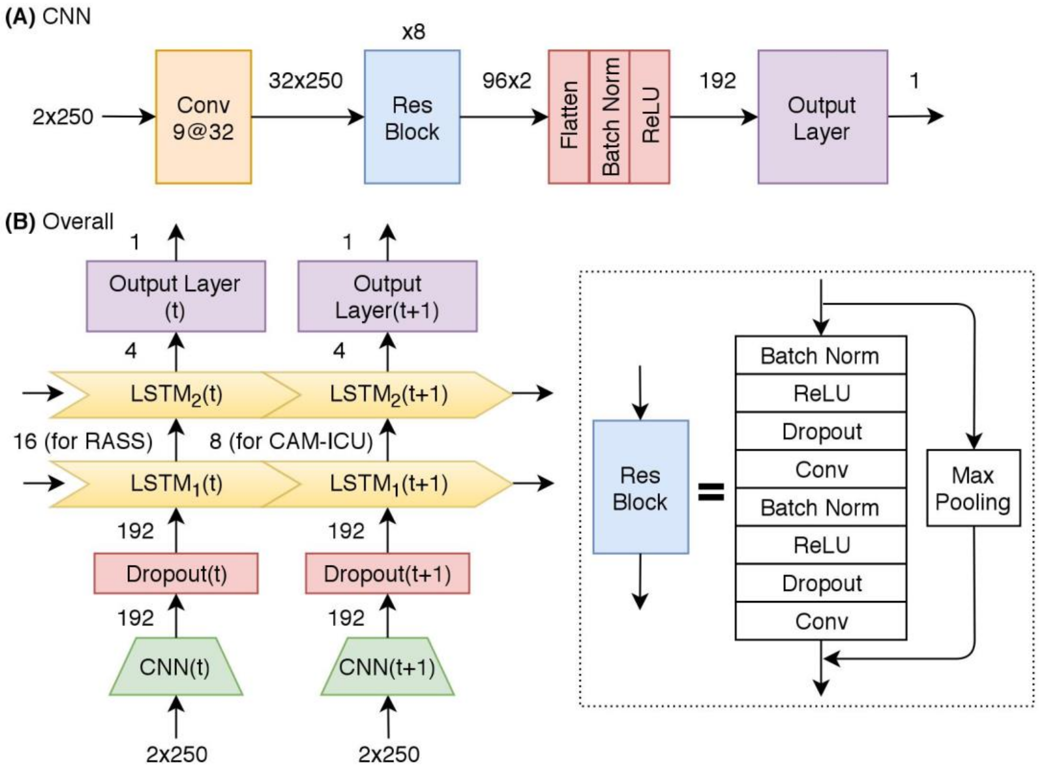

Supplementary Figure 9. Model architecture. (a) CNN architecture. The numbers such as "2x250" indicates there are 2 channels, and each channel has 250 sample points. "9@32" means in the convolutional layer, the filter size is 9 and the number of filters is 32. (b) The overall architecture. There are 2 layers of LSTM on top of CNN, which integrates the CNN outputs (not including the output layer in CNN) along time.

Supplementary Tables

Supplementary Table 1. Richmond agitation-sedation scale (RASS)[2]

Score	Term	Description
+4	Combative	Violent, immediate danger to staff
+3	Very agitation	Pulls on or removes tube(s) or catheter(s) or has aggressive behavior toward staff
+2	Agitated	Frequent non-purposeful movement or patient-ventilator dyssynchrony
+1	Restless	Anxious or apprehensive but movements not aggressive or vigorous
0	Alert and calm	
-1	Drowsy	Not fully alert, but has sustained (more than 10 seconds) awakening, with eye contact to voice
-2	Light sedation	Briefly (less than 10 seconds) awakens with eye contact to voice
-3	Moderate sedation	Any movement (but no eye contact) to voice
-4	Deep sedation	No response to voice, but some movement to physical stimulation
-5	Unarousable	No response to voice or physical stimulation

Supplementary Table 2. Confusion Assessment Method for the ICU (CAM-ICU)[3]

Feature	Criteria
Only proceed if RASS ≥ -3.<br>Otherwise not be able to assess. In this study, we set to delirium = YES.	
Feature 1:<br>Acute Onset or Fluctuating Course	Patient different than baseline, pre-hospital mental status.<br>OR<br>Patient with fluctuating mental status in past 24 hours by fluctuation of level of consciousness/sedation.
Feature 2:<br>Inattention	Letters attention test with >2 errors: Patient squeezes your hand when the letter A is spoken. Error is missing an A or squeezing without an A. Say C-A-S-A-B-L-A-N-C-A.
Feature 3:<br>Altered Level of Consciousness	RASS is not 0 (alert and calm).
Feature 4:<br>Disorganized Thinking	>1 Error questions and commands.
Delirium = Feature 1 AND Feature 2 AND (Feature 3 OR Feature 4)	

Supplementary Reference

1. Rennie, J. D. & Srebro, N. Loss functions for preference levels: Regression with discrete ordered labels. in *Proceedings of the IJCAI multidisciplinary workshop on advances in preference handling* 180–186 (Kluwer Norwell, MA, 2005).
2. Sessler, C. N. *et al.* The Richmond Agitation–Sedation Scale: validity and reliability in adult intensive care unit patients. *American journal of respiratory and critical care medicine* **166**, 1338–1344 (2002).
3. Ely, E. W. *et al.* Evaluation of delirium in critically ill patients: validation of the Confusion Assessment Method for the Intensive Care Unit (CAM-ICU). *Critical care medicine* **29**, 1370–1379 (2001).
